# Changes of Hemodynamic Parameters after Intradialytic Glucose Injection

**DOI:** 10.3390/nu15020437

**Published:** 2023-01-14

**Authors:** Longin Niemczyk, Katarzyna Romejko, Katarzyna Szamotulska, Daniel Schneditz, Stanisław Niemczyk

**Affiliations:** 1 Department of Nephrology, Dialysis and Internal Diseases, Medical University of Warsaw, 1a Banacha Street, 02-097 Warsaw, Poland; 2 Department of Internal Diseases, Nephrology and Dialysis, Military Institute of Medicine, 128 Szaserów Street, 04-141 Warsaw, Poland; 3 Department of Epidemiology and Biostatistics, Institute of Mother and Child, 17 a Kasprzaka Street, 01-211 Warsaw, Poland; 4 Otto Loewi Research Center, Division of Physiology, Medical University of Graz, Neue Stiftingtalstrasse 6/V, 8010 Graz, Austria

**Keywords:** chronic kidney disease, hemodialysis, hypotension, clinical experiment, arterial blood pressure, heart rate

## Abstract

Background: Intradialytic hypotension (IDH) is a frequent complication of hemodialysis (HD). Current methods of IDH prevention are insufficient. Methods: We analyzed the intradialytic time course of systolic (SBP), diastolic (DBP), mean arterial (MAP), pulse pressure (PP), and heart rate (HR) in a group of chronic kidney disease (CKD) patients. First, 30 min into HD, a 40% glucose solution was injected into the venous line of the extracorporeal circulation at a dose of 0.5 g/kg of dry weight. Pressures and HR were measured in frequent intervals. Relative volume overload was determined by bioimpedance spectroscopy. Results: Thirty-five participants were studied. SBP increased after 5, 10, and 20 min of glucose infusion. DBP increased after 2 and 3 h and also at the end of HD. PP increased after 5, 10, and 20 min of glucose infusion and fell after the 2nd and 3rd hour and also at the end of HD. MAP increased after 2 and 3 h of glucose injection and at the end of HD. Significant interactions of the time course of SBP, DBP, MAP, with HR at baseline and of the time course of PP with fluid overload were observed. Symptomatic hypotensive episodes were absent. Conclusions: Glucose infusions during HD prevent symptomatic IDH and do not cause severe hypertensive episodes.

## 1. Introduction

Intradialytic hypotension (IDH) is defined as a decline of blood pressure during hemodialysis (HD). IDH occurs in approximately 17% of hemodialysis treatments and is associated with elevated cardiovascular and all-cause mortality [1,2]. The definition of IDH is difficult because there is no clear blood pressure threshold below which hypotension during hemodialysis may be diagnosed. In several definitions of IDH, one or more of the following components should be found: a decline in blood pressure below a threshold/nadir, patient-reported symptoms, and medical intervention aimed at restoring blood volume [3]. The causes of intradialytic hypotension are multifactorial, but it seems that the main etiological factor is ultrafiltration-induced hypovolemia. In healthy individuals, volume removal intensifies compensatory mechanisms such as activation of the sympathetic nervous and the renin-angiotensin-aldosterone system and also enhances the vasopressin response. This results in a partial restoration of cardiac output and in an increase in total peripheral resistance to maintain the correct blood pressure. Because of numerous comorbidities in the group of dialysis patients, such as heart failure, coronary artery disease, diabetes, atherosclerosis, vascular calcification, and hypertension, the hemodynamic response due to hypovolemia may be insufficient to maintain a stable blood pressure. These states are characterized by impaired sympathetic nervous system activation, blunted vasopressin response, decreased total peripheral resistance, and lower cardiac output [4]. Additional factors contributing to IDH are poor nutritional status, female sex, and high body mass index (BMI) [5]. Older age may also contribute to IDH although Capuano’s study did not confirm a difference in the incidence of IDH between younger and older patients, which is explained by lower ultrafiltration requirements as well as smaller osmolality changes during hemodialysis [6]. On the other hand, Bossola found that the incidence of IDH increases with dialysis vintage [7]. Intradialytic weight gain above 3 kg, imprecise assessment of dry weight, large volume ultrafiltration, and a decrease in serum osmolality during hemodialysis contribute to decline of blood pressure [8]. The consequence of IDH is reduced tissue perfusion, for example, in the myocardium impairing systolic function, causing myocardial stunning and thus increasing cardiovascular mortality [9]. However, hypoperfusion and the risk for ischemia is not limited to the heart. Hypoperfusion affects the brain, the hepato-splanchnic system, and the residual function of the kidneys. Decreased cerebral oxygenation leads to ischemic brain injury and dementia. Prolonged hepato-splanchnic vasoconstriction may lead to gut ischemia and translocation of endotoxin to blood, increasing inflammation and the development of protein energy wasting [10,11]. IDH also causes kidney ischemia and accelerates the loss of residual renal function, which is unfavorable especially for patients with residual diuresis. Frequent IDH episodes are related with higher all-cause mortality [12]. Symptoms of IDH such as muscle cramps, nausea, vomiting, dizziness, fainting, abdominal pain are stressful for patients and worsen their quality of life. Methods to prevent IDH include the reduction of interdialytic weight gain by lowering dietary sodium and fluid intake. Among other preventive procedures, the appropriate assessment of volume status and dry weight, increasing the frequency and/or the duration of hemodialysis sessions, lowering the dialysate temperature, and reducing ultrafiltration and antihypertensive drugs are enumerated [13,14,15]. The occurrence of IDH may require infusion of fluids such as crystalloids or colloids [16]. Glucose solutions are of special interest because, contrary to NaCl solutions, the osmotic effect of glucose solutions dissipates as soon as glucose is disposed and metabolized. However, because of the high prevalence of type 2 diabetes mellitus in chronic kidney disease patients, the question also arises whether the infusion of concentrated glucose is feasible and beneficial during hemodialysis. The effect of concentrated glucose infusion on glucose and insulin kinetics in chronic and stable hemodialysis patients with and without type 2 diabetes mellitus during hemodialysis has been reported elsewhere [17,18]. The hemodynamic effect of such infusions was, however, incompletely studied.

The purpose of this study was to evaluate the hemodynamic response after intravenous injection of glucose in a mixed group of maintenance dialysis patients. We estimated whether intravenous administration of glucose would be beneficial to prevent a fall of blood pressure during hemodialysis.

## 2. Methods

### 2.1. Design

This is the continuation of a study on glucose and insulin kinetics performed in chronic and stable hemodialysis patients during their regular dialysis treatment using the infusion of concentrated glucose solution, which has been reported elsewhere [17,18]. The time course and the magnitude of the perturbation was comparable to that of an intravenous glucose tolerance test, but it was completed during hemodialysis. This perturbation not only has metabolic effects that were analyzed and discussed previously but also the significant hemodynamic and blood volume effects analyzed and discussed in this article and in companion papers published in the same journal [19,20].

We performed an experimental study in the group of patients with chronic kidney disease (CKD) treated with hemodialysis. After 30 min of the beginning of hemodialysis, a 40% glucose solution was injected into the venous line of extracorporeal circulation at a dose of 0.5 g per kg of dry weight comparable to the dose of an intravenous glucose tolerance test. Systolic and diastolic arterial blood pressures (SBP and DBP) and heart rate (HR) were measured by an automatic oscillometric method and an inflatable arm-cuff located on the upper arm of the contra-lateral side of the vascular access, then manually recorded in the database. Measurements were taken at the beginning of HD (30 min before glucose bolus injection, T_-30_); at the time of glucose injection (T_0_); and 5, 10, 20, 30, 60, 120, and 180 min after glucose injection (T_5_, T_10_, T_20_, T_30_, T_60_, T_120_, and T_180_); and at the end of HD (T_end_). Pulse pressure (PP) was calculated from the difference of SBP and DBP. Mean arterial pressure (MAP) was calculated as MAP = DBP + PP/3.

Blood samples for measuring glucose, sodium, and potassium concentrations were drawn from the arterial blood line of the extracorporeal circulation 30 min before the start of glucose injection (T_-30_), at the time of glucose injection (T_0_), and 5, 10, 20, 30, and 60 min after glucose injection (T_5_, T_10_, T_20_, T_30_, and T_60_).

The experiment started in the morning hours. Patients were asked not to eat and drink more than 3 h before HD and to assume a supine body position for the duration of the test. In patients with diabetes, insulin administration was withheld for 8 h before starting the study.

Bioimpedance spectroscopy was performed at the day of the experiment before HD with the use of a body composition monitor (BCM, Fresenius Medical Care, Bad Homburg v.d.H., Germany) after 5 min supine rest to quantify the relative extracellular volume overload (Vo%). Electrodes were placed on one hand and one foot of the contra-lateral side of the vascular access in a tetrapolar configuration.

### 2.2. Patients

The study sample consisted of 35 CKD patients treated with HD in the Dialysis Department of the Military Institute of Medicine, Warsaw, Poland. The inclusion criteria of the study were age above 18 years, HD treatment for more than 3 months using a peripheral arterio-venous access with an access flow of more than 600 mL/min, and a minimum extracorporeal blood flow of 250 mL/min. Adequate dialysis dose was quantified as Kt/V > 1.2. Clinical signs of infection, symptomatic anemia, wasting disease, hormonal therapy, diabetogenic drug prescription, and the lack of agreement to participate in the study were the exclusion criteria. More information on the material and methods can be found in the companion papers to this work [17,18].

### 2.3. Statistical Analysis

Results are presented as means ± standard deviations (SD) for normally distributed data or medians and interquartile ranges (IQR) for non-normally distributed variables. The Kolmogorov–Smirnov test was performed for evaluating distributions for normality. For comparison of observations between time points, the paired *t*-test was applied. To investigate changes of continuous parameters over time and to compare the time course of these parameters between different subgroups, MANOVA for repeated measurements was used. Mauchly’s test for sphericity and Greenhouse–Geisser adjustment were employed if necessary. A *p*-value < 0.05 was considered to be statistically significant. Statistical analyses were run in IBM SPSS ver. 25.

## 3. Results

The study population consisted of 12 women (34.3%) and 23 men (65.7%). The average age was 61.2 ± 13.6 years, and the mean body mass index (BMI) was 28.2 ± 5.2 kg/m^2^. Type 2 diabetes mellitus was diagnosed in 14 patients (40%). Median dialysis vintage was 2 years, and the mean ultrafiltration volume was 2.8 L with the IQR of 1.8–3.0 L. The mean SBP, DBP, and MAP at the beginning of dialysis (T_30_) were, respectively, 132.5 ± 23.0, 61.8 ± 14.8, and 85.4 ± 14.1 mmHg. HR was 69.5 ± 10.8 beats per minute (bpm), and PP was 70.7 ± 23.6 mmHg at the beginning of dialysis. The median serum glucose concentration at T_-30_ was 102 mg/dL (IQR 90 to 128 mg/dL); mean serum sodium and potassium concentrations were 144.5 ± 3.0 mmol/L and 5.0 ± 0.8 mmol/L. Relative volume overload (Vo%) before dialysis was 12.4% with IQR of 3.7–16.4%. Clinical data of the studied sample are presented in Table 1. 

**Figure 1:**
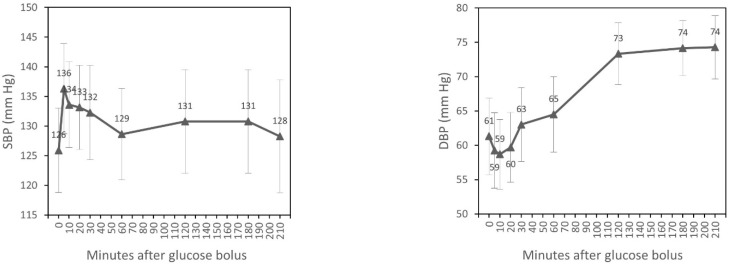

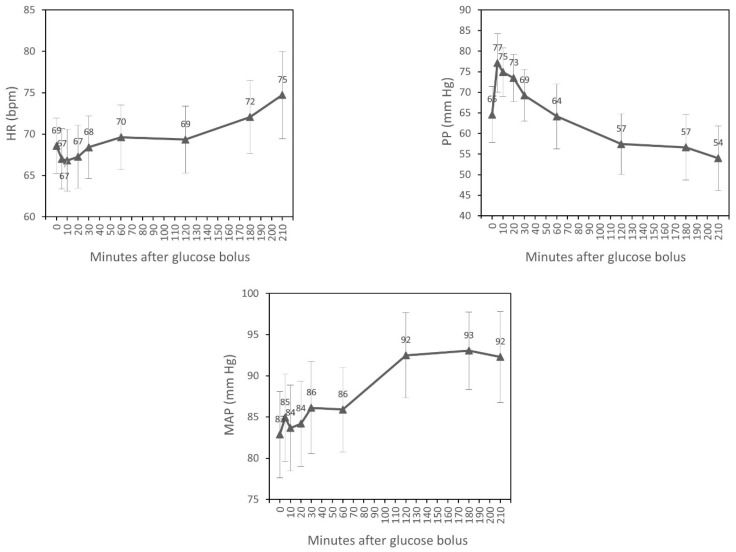
Time course of hemodynamic parameters during hemodialysis (estimated marginal means, 95% CI ).

SBP significantly increased after 5, 10, and 20 min of glucose bolus (*p* < 0.001, *p* = 0.003, *p* = 0.023, respectively) compared to the time of glucose injection (T_0_). A statistically significant rise of DBP after 2 and 3 h (*p* < 0.001, *p* < 0.001) and also at the end of dialysis (*p* < 0.001) was observed. HR increased significantly after 3 h of the glucose bolus (*p* = 0.027) and at the end of the experiment (*p* = 0.002). PP increased after 5, 10, and 20 min of the glucose bolus (*p* < 0.001, *p* < 0.001, *p* = 0.002, respectively) and fell significantly after second hour (*p* = 0.037) and third hour (*p* = 0.019) and also at the end of the experiment (*p* = 0.002). We observed a statistically significant increase of MAP after 2 and 3 h of glucose injection (*p* < 0.001, *p* = 0.001) and at the end of the experiment (*p* = 0.003) (Table 2). However, MANOVA for repeated measurements showed overall changes in the course over time only for DBP, HR, PP, and MAP (Figure 1).

### 3.1. Systolic Blood Pressure

In univariate analyses, we found that the time course of SBP during HD differed between diabetic and non-diabetic patients (*p* = 0.009). In individuals with diabetes, SBP increased after 30 min of glucose infusion, contrary to patients without diabetes, where we observed the systematic fall of SBP after 5 min of glucose bolus to the end of HD. We also found an interaction of the time course of SBP with HR at T_-30_. SBP increased after one hour of glucose infusion in patients with HR < 60 bpm at the beginning of HD, contrary to those with HR 60–100 bpm in T_-30_ of the experiment, where we observed the systematic fall of SBP during HD (*p* = 0.001) (Table 3).

Additionally, patients with SBP above 140 mmHg at the beginning of HD had on average higher SBP throughout HD than participants with SBP ≤ 140 mmHg at baseline (*p* < 0.001).

**Figure 2:**
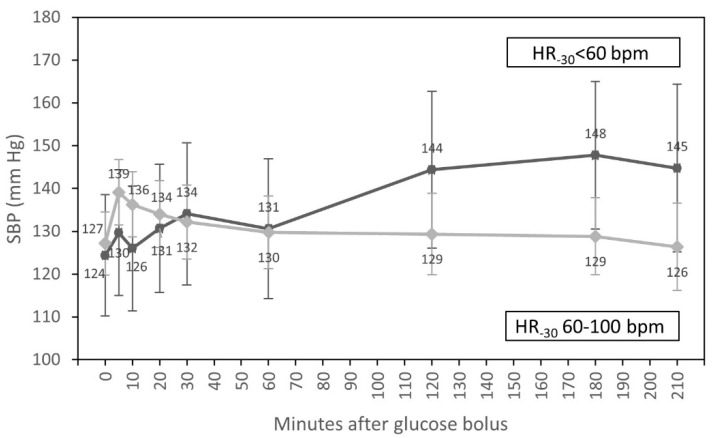
Course of systolic blood pressure (estimated marginal means, 95% CI) for groups with HR < 60 bpm and HR from 60 to 100 bpm at the beginning of hemodialysis, adjusted for diabetic status and systolic blood pressure at the start of HD.

In multivariate analysis, only an interaction of the time course of SBP with HR at T_-30_ (*p* = 0.014) (Figure 2) and differences in SBP throughout the whole HD between participants with higher and lower values of SBP at T_-30_ (*p* = 0.001) were statistically significant.

### 3.2. Diastolic Blood Pressure

In univariate analyses, the time course of DBP was influenced by HR at T_-30_ (*p* = 0.005) and by sex (*p* = 0.034). Patients with HR from 60 to 100 bpm at baseline had higher DBP than participants with HR < 60 bpm up to 60 min after the glucose infusion. Two hours after glucose injection, DBP of patients with HR < 60 bpm at the beginning of dialysis increased, and readings levelled off in patients with low and normal HR before dialysis. In addition, men had higher DBP than women up to 60 min after the glucose bolus. Two hours after glucose injection, DBP readings levelled off in both sexes (Table 4).

Additionally, patients with diabetes had systematically lower DBP throughout the whole HD compared to those without diabetes (*p* = 0.009).

**Figure 3:**
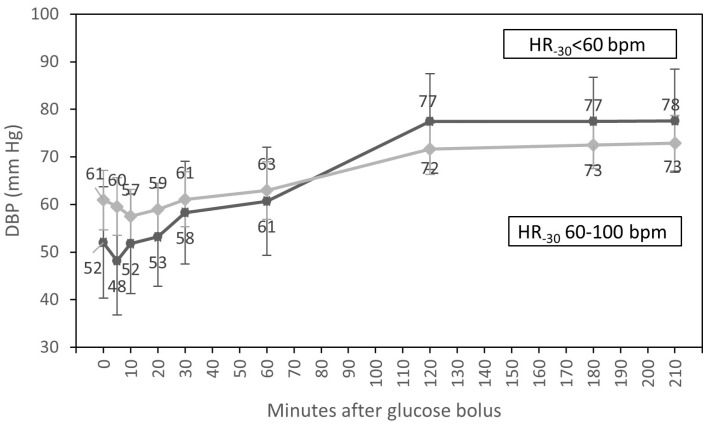
Course of diastolic blood pressure (estimated marginal means, 95% CI) for groups with HR <60 bpm and HR from 60 to 100 bpm at the beginning of hemodialysis, adjusted for diabetic status and sex.

In multivariate analysis, only an interaction of the time course of DBP with HR at T_-30_ (*p* = 0.017) (Figure 3) and differences in DBP throughout the whole HD session for participants with diabetes compared to patients without diabetes (*p* = 0.028) were statistically significant.

### 3.3. Heart Rate

In univariate analyses, the course of heart rate over time was not affected by any of the measured factors. However, we found that HR in overweight patients was systematically higher compared to participants with normal weight (*p* = 0.020). We also observed that individuals with HR < 60 bpm at the beginning of dialysis had lower HR throughout the whole HD compared to those with HR from 60 to 100 bpm at baseline (*p* < 0.001). Additionally, during the experiment, diabetic patients had significantly lower HR than those without diabetes (*p* = 0.006) (Table 5). 

In multivariate analysis, only the differences in HR throughout the whole treatment for participants with HR < 60 bpm at the beginning of dialysis compared to participants with HR from 60 to 100 bpm at baseline (*p* = 0.001) and the differences in HR throughout the whole HD for participants with diabetes compared to patients without diabetes (*p* = 0.015) were statistically significant.

### 3.4. Pulse Pressure 

In univariate analyses, an interaction of the time course of PP and diabetic status and an interaction of the time course of PP and fluid overload were observed. We found that the fall of PP after 60 min of the glucose infusion was significantly lower in diabetic patients in comparison to individuals without diabetes (*p* = 0.047). We also observed that the decrease in PP after 20 min of glucose infusion was significantly lower in patients with fluid overload > 15% compared to subjects with a volume overload of ≤ 15% (*p* = 0.005) (Table 6).

Additionally, PP was higher in diabetic patients compared to individuals without diabetes throughout the whole dialysis session (*p* = 0.009). We also found systematic differences in the time course of PP between patients with elevated SBP > 140 mmHg at the beginning of dialysis compared to patients with SBP ranging from 90 to 140 mmHg. Those with higher SBP at baseline had significantly higher PP during dialysis compared to participants with low SBP ranging from 90 to 140 mmHg at baseline (*p* < 0.001). Patients with relative fluid volume overload > 15% at the beginning of dialysis also had higher PP throughout the experiment compared to participants with a relative volume overload ≤ 15% (*p* = 0.021).

**Figure 4:**
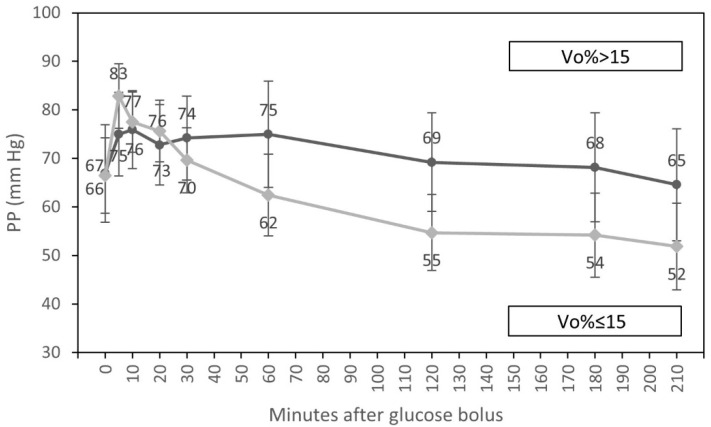
Course of pulse pressure (estimated marginal means, 95% CI) for groups with low (<15%) and elevated (≥15%) relative volume overload at the beginning of hemodialysis, adjusted for diabetic status and systolic blood pressure at the start of HD.

In multivariate analysis, only an interaction of the time course of PP with fluid overload at the beginning of dialysis (*p* = 0.006) (Figure 4), differences in PP throughout the whole treatment session for participants with diabetes compared to patients without diabetes (*p* = 0.012), and systematic differences in PP during the experiment for participants with higher vs. lower SBP at baseline (*p* < 0.001) were statistically significant.

### 3.5. Mean Arterial Pressure

In univariate analyses, an interaction of the time course of MAP with HR at the beginning of HD and an interaction of the time course of MAP with diabetes were observed. MAP increased significantly between one and two hours after glucose infusion in participants with HR < 60 bpm compared to those with HR 60–100 bpm at the beginning of dialysis (*p* = 0.001). We also observed differences in MAP increase after 60 and 120 min of glucose infusion between patients with and without diabetes and where the increase of MAP was significantly higher in diabetic individuals (*p* = 0.020) (Table 7).

**Figure 5:**
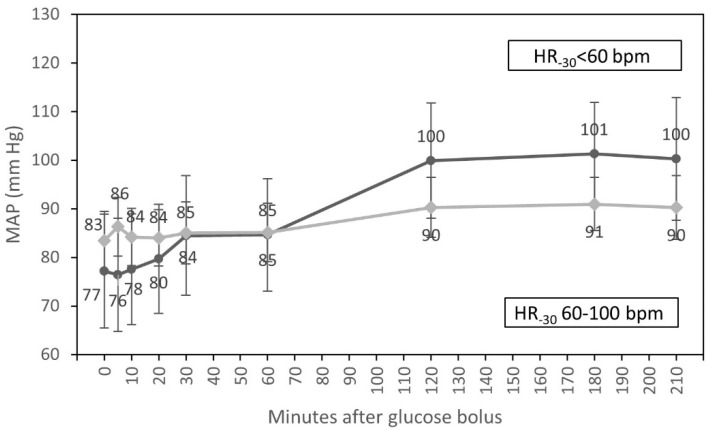
Course of mean arterial pressure (estimated marginal means, 95% CI) for groups with HR < 60 bpm and HR from 60 to 100 bpm at the beginning of hemodialysis, adjusted for diabetic status.

In multivariate analysis, only an interaction of the time course of MAP with HR at T_-30_ (*p* = 0.005) was statistically significant (Figure 5).

## 4. Discussion

All studies were completed without symptomatic signs of IDH. Our experiment confirms that glucose infusion during HD prevents symptomatic IDH episodes. 

The decline of blood pressure during HD is associated with elevated mortality, myocardial stunning, ischemic brain injury, increase of inflammatory processes, and kidney or gut ischemia [9,10]. However, it is crucial not only to treat IDH but also to prevent hypertensive episodes during HD. Despite complications of hemodynamic dysregulation during HD, there are also psychological aspects of IDH. Episodes of blood pressure falls and symptoms of hypotension such as nausea, vomiting, abdominal pain, and dizziness are very stressful for patients and worsen their quality of life. This justifies the search for new therapeutic methods that may prevent the fall of blood pressure during HD. Methods to prevent IDH include the reduction of interdialytic weight gain, the proper assessment of dry weight before HD, the reduction of ultrafiltration volume and rate, and the reduction of hypertensive drugs. Higher dialysate calcium concentration augments myocardial contractility and minimizes the decline of blood pressure during HD [21]. Another preventive method of IDH is the restriction of intradialytic food intake. Food ingestion increases hepato-splanchnic blood flow, which may result in postprandial hypotension [22]. It has been well-established for more than 40 years that cooling the dialysate reduces IDH frequency [23,24]. Vasoconstrictors such as midodrine or vasopressin have also been used to increase arterial blood pressures during HD and to prevent episodes of IDH [25,26]. An additional strategy to prevent IDH is by increasing vascular refilling with the use of higher dialysate sodium concentrations. However, this method is not without side effects, as the addition of extra sodium chloride increases thirst and thereby increases intradialytic weight gain, which defeats the purpose of reducing ultrafiltration requirements [27]. Intravenous administration of glucose seems to have more beneficial aspects compared to saline solution. It provides fluid volume and transient tonicity without extra sodium load. In a previous study done in non-diabetic patients and using a continuous beat-to-beat arterial blood pressure measuring system, glucose administration led to a moderate and transient rise in arterial pressures and HR, which dissipated one hour after injection [28]. Despite the small study sample, it may be assumed that even large amounts of glucose do not cause long-lasting hemodynamic effects because glucose is efficiently eliminated by ongoing hemodialysis [28]. In the current study, we decided to infuse glucose to diabetic patients during hemodialysis as well. This can be done because excess glucose is rapidly removed by hemodialysis in diabetic patients. Indeed, there is only little difference in glucose disposal between diabetic and non-diabetic patients during hemodialysis [17].

In our study, we found that on average the infusion of glucose caused higher SBP from 5 to 20 min after glucose infusion, followed by a slight (statistically insignificant) decrease lasting until the end of dialysis. This increase is essentially caused by the osmotic action of glucose expanding the circulating blood volume and which is studied in a companion paper [20]. Changes of DBP after glucose infusion were also observed, but DBP increased significantly between 60 and 120 min of HD, long after the direct glucose effect had disappeared, and remained higher until the end of dialysis, similarly to MAP. We also observed that HR increased significantly after 2 h of dialysis (Table 2, Figure 1).

Patients with CKD treated with HD are prone to have fluctuations in blood pressure values depending on hemodialysis schedule. Blood pressure tends to be highest in predialysis period, then decreases during HD, and after that rises again in the next interdialytic time period. The average decline of blood pressure during HD is about 28 to 40 mmHg [4]. Systolic blood pressure in patients treated with HD has a “U” shape with the fall during HD.

The results of our report show that glucose infusion in the large dose of 0.5 g/kg of dry weight prevents a serious fall of blood pressure during HD. When we analyzed the relationship between the time course of SBP and selected clinical and dialysis parameters, we found an interaction of time course of SBP with HR at T_-30_ (*p* = 0.014) and higher SBP throughout the whole HD for those with higher SBP at T_-30_ (*p* = 0.001). The infusion of glucose did not cause a long-lasting rise of SBP in patients with normal HR values at T_-30_, but on the other hand, it caused an increase of SBP after 60 min in those with HR < 60 bpm at T_-30_. Thus, we may state that the glucose infusion prevents a serious fall of SBP in those with HR < 60 bpm, who may not be able to compensate the fall of blood pressure with an adequate increase in HR, which is one of the physiologic responses to hypovolemia (the other being peripheral vasoconstriction). However, this physiologic response is often impaired in CKD because of frequent cardiovascular complications including heart failure [29]. The use of beta-blockers with negative inotropic and chronotropic effects may also contribute to IDH by lowering contractility and heart rate. The impaired autonomic response in this group of patients including diabetic participants is also responsible for insufficient autonomic response to blood pressure decrease, which leads to arteriolar vasodilatation, bradycardia, and IDH.

In the course of the experiment, we found that ten patients had at least one episode of SBP < 100 mmHg, and among them, three participants had at least one episode of SBP < 90 mmHg. Patients with at least one episode of SBP < 100 mmHg during HD had significantly lower SBP at the beginning of dialysis compared to those with SBP ≥ 100 mmHg (*p* < 0.001). They also had lower DBP in each moment of HD compared to participants with SBP ≥ 100 mmHg. Patients with at least one episode of SBP < 100 mmHg during HD were older, had lower BMI, and had lower HR at the beginning of HD compared to those with SBP ≥ 100 mmHg. Participants with episodes of SBP < 100 mmHg and those with SBP ≥ 100 mmHg during HD did not differ in terms of sex or diabetes. Despite the fact that ten patients had at least one episode of SBP < 100 mmHg, these episodes were not diagnosed as IDH. Participants with SBP < 100 mmHg during HD had lower SBP at the beginning of HD, they did not have clinical symptoms of hypotension, and they did not require medical intervention throughout the whole HD. However, we also observed an increase in arterial blood pressure one hour after glucose infusion in patients with baseline HR_-30_ < 60 bpm: 14 participants had at least one episode of an increase of SBP > 20 mmHg in relation to SBP_-30_. The rise of SBP was noticed immediately after glucose infusion, and SBP remained high for the following two hours. Participants with a rise in SBP > 20 mmHg during HD had higher DBP throughout the whole HD, they were older than those without such an increase, they had higher glucose and sodium concentrations at the beginning of HD, they had lower ultrafiltration volumes, and had increased fluid overload compared to individuals without episodes of SBP rise > 20 mmHg. Overall, 57% of participants with episodes of SBP rise > 20 mmHg were diabetic patients. Despite the rise of SBP > 20 mmHg during HD, participants did not report clinical symptoms of hypertension and also did not require medical intervention or additional hypotensive treatment throughout the whole HD. We therefore conclude that glucose infusion did not cause the severe hypertensive episodes in the study population.

We also found that patients with HR < 60 bpm at baseline had lower DBP in comparison to participants with HR from 60 to100 bpm for the 60 min period following the glucose infusion. After one hour of glucose infusion, DBP in patients with HR < 60 bpm at baseline rose and remained elevated compared to participants with HR from 60 to100 bpm (Figure 3). However, the overwhelming majority of our low-HR patients were diabetics (71.4%). Diabetic patients frequently have an impaired autonomic response to hypotension compared to healthy individuals. Autonomic mechanisms such as the inotropic response of the myocardium or the vasoconstriction of arterioles may be insufficient in diabetes to maintain an adequate blood pressure. In our study, we found that the glucose infusion during HD in patients with HR < 60 at T_-30_ prevented a potential fall in DBP after one hour of the glucose infusion. It may be especially important for those with HR < 60 bpm, where the compensatory mechanisms of blood pressure control connected with HR variability may be impaired [19]. Moreover, we observed that patients with diabetes had lower DBP throughout the whole treatment session compared to those without diabetes (*p* = 0.028). Our observation that patients with diabetes and HR < 60 bpm at T_30_ experienced a rise in DBP after 60 min of the glucose infusion without serious side effects is crucial to predict that this may be a potential preventive method of IDH episodes in the diabetic dialysis population.

Heart failure occurs in up to 65–70% of patients with CKD [30]. Both systolic and diastolic functions may be impaired. Left ventricular diastolic dysfunction is observed even in early stages of kidney function decrease [31]. Decreased cardiac output and insufficient sympathetic compensation may result in an excessive fall in blood pressure. PP, also known as pulse amplitude, is defined as the difference between systolic and diastolic blood pressures. While the chronic increase indicates reduced arterial vascular compliance, the acute increase (or decrease) indicates an acute increase (or decrease) in stroke volume [32,33]. In our report, PP increased significantly and remained elevated after the glucose infusion, when we also observed the rise of SBP. The drop of PP was reported from the second hour to the end of HD because of the rise in DBP from the second hour to the end of HD (Table 2). The acute changes are essentially explained by glucose-induced blood volume expansion and by the resulting increase in cardiac output, studied in previous and companion publications [20,28]. We also found significantly higher PP in diabetic patients compared to non-diabetic individuals (*p* = 0.012). This chronic difference is essentially explained by stiff arteries found in diabetic subjects. The meta-analysis by Kodama based on almost 58,000 patients showed that increased PP is associated with future cardiovascular risk in diabetic individuals [34]. Dillinger also reported that elevated PP in diabetic patients was associated with increased all-cause mortality [35]. The most probable reasons for elevated PP in diabetic patients are accelerated atherosclerosis and aortic stiffness [36]. We also found that the decrease in PP after glucose infusion was significantly lower in volume-expanded participants (*p* = 0.006) (Figure 4). CKD and hemodialysis treatment are the states with a wide range of PP values. Several studies found positive associations between high PP and increased mortality in CKD [37,38,39]. Additionally, in our report, we found that patients with higher SBP at baseline had significantly higher PP during the whole treatment session compared to those with lower SBP at the beginning of dialysis (*p* < 0.001). An isolated increase in SBP already leads to an increase in PP. Isolated systolic hypertension is diagnosed in around 65% of hypertensive elderly patients and is connected with atherosclerosis and arterial stiffness [40]. Cardiovascular complications, including atherosclerosis, are the main cause of morbidity and mortality in CKD patients. As a high PP is related with elevated mortality in CKD, and as higher PP values are associated with diabetes and increased SBP at the beginning of dialysis, as shown in this study, the estimation of PP should be considered as a routine measurement to improve the diagnostics and the treatment procedures in this vulnerable population of CKD patients treated with HD. 

We found that MAP increased significantly between one and two hours after bolus injection. In multivariate analysis, an interaction of the time course of MAP with HR at T_30_ was statistically significant (*p* = 0.005) (Figure 5). One hour after the infusion of the glucose, we observed an increase in MAP in patients with HR < 60 bpm at T_-30_. Participants with HR 60–100 bpm at T_-30_ did not have a significant increase in MAP. In participants with HR < 60 bpm at T_-30_ who may not be able to compensate for a fall of blood pressure with an increase of HR, the infusion of glucose may prevent the fall of arterial blood pressure. The majority of patients with HR < 60 bpm at T_-30_ were diabetic. Short infusions of glucose solution increase osmolality to a greater extent in diabetic participants, who have higher serum glucose concentrations also before the glucose administration. The elimination of serum glucose during HD lasts somewhat longer in diabetic participants compared to those without diabetes [17]. This could be an explanation for the increase of MAP after one hour of the glucose infusion in diabetic participants.

The small sample size is one limitation of our study. A much larger number of patients would be required to analyze effects of age, comorbidities, and medication. The high age of the study population could be considered as a limitation as well, especially as the effect of age on IDH remains unclear.

## 5. Conclusions

IDH is associated with increased cardiovascular and all-cause mortality, and that is why it is necessary to examine new methods that prevent a fall in blood pressure during HD but do not lead to volume overload and do not cause a severe rise in arterial blood pressure. Glucose infusion seems to combine those advantages. From our study, we may conclude that glucose infusion prevents episodes of IDH during HD and does not cause severe hypertensive episodes. However, more studies on delayed rise of arterial blood pressure after glucose infusion injection in patients with low HR and/or diabetes are needed.

## Figures and Tables

**Table 1:** Clinical and dialysis characteristics of the studied sample.

	n = 35
Age (years), mean ± SD	61.2 ± 13.6
Males (%)	65.7
Diabetes (%)	40.0
Body mass index (kg/m^2^), mean ± SD	28.2 ± 5.2
SBP_-30_ (mm Hg), mean ± SD	132.5 ± 23.0
DBP_-30_ (mm Hg), mean ± SD	61.8 ± 14.8
MAP_-30_ (mm Hg), mean ± SD	85.4 ± 14.1
HR_-30_ (bpm), mean ± SD	69.5 ± 10.8
PP_-30_ (mm Hg), mean ± SD	70.7 ± 23.6
Serum sodium_-30_ (mmol/L), mean ± SD	144.5 ± 3.0
Serum potassium_-30_ (mmol/L), mean ± SD	5.0 ± 0.8
Serum glucose_-30_ (mg/dL), median (IQR)	102 (90–128)
Relative volume overload (%)	12.4 (3.7–16.4)
Ultrafiltration volume (L), mean ± SD	2.8 (1.8–3.0)
Dialysis vintage (years), median (IQR)	2 (1–3)

SBP_-30_, systolic blood pressure at the beginning of dialysis; DBP_-30_, diastolic blood pressure at the beginning of dialysis; MAP_-30_, mean arterial pressure at the beginning of dialysis; HR_-30_, heart rate at the beginning of dialysis; bpm, beats per minute; PP_-30_, pulse pressure at the beginning of dialysis;.

**Table 2:** Hemodynamic parameters during hemodialysis.

T	N	SBP (mm Hg)		DBP (mm Hg)		HR (bpm)		PP (mm Hg)		MAP (mm Hg)	
		Mean ± SD (Range)	*p*-Value *	Mean ± SD (Range)	*p*-Value *	Mean ± SD (Range)	*p*-Value *	Mean ± SD (Range)	*p*-Value *	Mean ± SD (Range)	*p*-Value *
T_-30_	35	132.5 ± 23.0 (91–170)	-	61.8 ± 14.8 (30–92)	-	69.5 ± 10.8 (46–94)	-	70.7 ± 23.6 (41–130)	-	85.4 ± 14.1 (61–115)	-
T_0_	35	125.9 ± 20.6 (84–161)	ref.	61.3 ± 16.2 (36–104)	ref.	68.6 ± 9.8 (46–87)	ref.	64.6 ± 19.7 (39–120)	ref.	82.8 ± 15.2 (57–122)	ref.
T_5_	35	136.3 ± 22.2 (92–179)	**<0.001**	59.2 ± 16 (33–86)	0.062	67. ± 10.7 (48–95)	0.106	77.1 ± 20.7 (20–121)	**<0.001**	84.9 ± 15.5 (64–120)	0.132
T_10_	35	133.6 ± 21.1 (100–175)	**0.003**	58.7 ± 14.9 (31–93)	0.120	66.8 ± 10.7 (50–93)	0.166	74.9 ± 17.3 (46–116)	**<0.001**	83.7 ± 15.1 (55–111)	0.616
T_20_	35	133.2 ± 20.7 (94–169)	**0.023**	59.7 ± 14.8 (32–86)	0.329	67.3 ± 11.0 (49–100)	0.241	73.5 ± 16.7 (51–114)	**0.002**	84.2 ± 15.0 (60–123)	0.471
T_30_	35	132.3 ± 23.1 (93–180)	0.074	63. ± 15.6 (38–100)	0.300	68.4 ± 11.0 (49–90)	0.867	69.3 ± 18.3 (44–122)	0.098	86.1 ± 16.3 (52–121)	0.116
T_60_	35	128.7 ± 22.5 (75–169)	0.400	64.5 ± 16. (40–98)	0.078	69.6 ± 11.4 (47–93)	0.266	64.1 ± 22.9 (20–124)	0.882	85.9 ± 14.9 (52–122)	0.110
T_120_	35	130.8 ± 25.4 (75–181)	0.186	73.3 ± 13.0 (40–108)	**<0.001**	69.3 ± 11.7 (45–98)	0.538	57.4 ± 21.4 (34–115)	**0.037**	92.5 ± 15.1 (71–120)	**<0.001**
T_180_	35	130.8 ± 25.4 (93–181)	0.246	74.2 ± 11.6 (57–106)	**<0.001**	72.1 ± 12.8 (44–99)	**0.027**	56.6 ± 23.1 (29–112)	**0.019**	93.0. ± 13.7	**0.001**
T_end_	35	128.3 ± 27.7 (77–170)	0.579	74.3 ± 13.4 (53–102)	**<0.001**	74.7 ± 15.3 (45–112)	**0.002**	54.0 ± 22.9 (24–115)	**0.002**	92.3 ± 16.1 (61–120)	**0.003**

* in comparison to T_0_. SBP, systolic blood pressure; DBP, diastolic blood pressure; HR, heart rate; PP, pulse pressure; MAP, mean arterial pressure; *p*-values < 0.05 are marked in bold.

**Table 3:** Dynamics of systolic blood pressure during hemodialysis in relation to selected clinical and dialysis parameters.

	Time Effect *p*-Value	Between Effect			Interaction with Time *p*-Value
		Difference from the Reference Value		*p*-Value	
		Mean	95% CI		
Males versus females	0.264	−1.6	−15.2; 12.0	0.808	0.829
Age ≥ 65 versus <65 (years)	0.212	−4.7	−17.5; 8.2	0.464	0.898
Body mass index	0.213			0.691	0.515
25.0–29.9 versus ≤24.9 (kg/m^2^)		6.3	−10.2; 22.7		
≥30.0 versus ≤24.9 (kg/m^2^)		5.9	−10.2; 22.1		
Diabetes—presence versus absence	0.262	3.7	−9.5; 16.8	0.574	**0.009**
SBP_-30_ >140 versus ≤140 (mm Hg)	0.211	20.6	9.9; 31.3	**<0.001**	0.930
HR_-30_ < 60 versus 60–100 (bpm)	0.165	6.7	−9.3; 22.6	0.401	**0.001**
Vo% >15 versus ≤15 (%)	0.332	−4.3	−13.6; 5.0	0.172	0.177
Ultrafiltration volume ≥ 2.5 (L)	0.197	−2.0	−15.2; 11.2	0.758	0.129
Serum sodium_-30_ > 145 versus ≤145 (mmol/L)	0.211	2.3	−10.6; 15.2	0.715	0.420
Serum potassium_-30_ > 5.0 versus ≤5.0 (mmol/L)	0.225	2.0	−10.9; 15.0	0.719	0.463
Dialysis vintage	0.078			0.486	0.666
0, 1 versus 2 (years)		−10.6	−28.5; 7.3		
≥3 versus 2 (years)		−5.7	−23.6; 12.2		

SBP_-30_, systolic blood pressure at the beginning of dialysis (baseline); HR_-30_, heart rate at the beginning of dialysis (baseline); Vo%, relative volume overload; *p*-values < 0.05 are marked in bold.

**Table 4:** Dynamics of diastolic blood pressure during hemodialysis in relation to selected clinical and dialysis parameters.

	Time Effect *p*-Value	Between Effect			Interaction with Time *p*-Value
		Difference from the Reference Value		*p*-Value	
		Mean	95% CI		
Males versus females	**<0.001**	5.8	−3.2; 14.8	0.201	**0.034**
Age ≥ 65 versus <65 (years)	**<0.001**	−6.8	−15.3; 1.6	0.109	0.956
Body mass index	**<0.001**			0.344	0.538
25.0–29.9 versus ≤24.9 (kg/m^2^)		6.8	−4.1; 17.7		
≥30.0 versus ≤24.9 (kg/m^2^)		0.3	−10.4; 11.1		
Diabetes—presence versus absence	**<0.001**	−11.0	−19.1; −3.0	**0.009**	0.078
SBP_-30_ >140 versus ≤140 (mm Hg)	**<0.001**	0.6	−8.2; 9.4	0.893	0.805
HR_-30_ < 60 versus 60–100 (bpm)	**<0.001**	−6.6	−17.4; 4.0	0.214	**0.005**
Vo% >15 versus ≤15 (%)	**<0.001**	−4.3	−13.6; 5.0	0.351	0.548
Ultrafiltration volume ≥ 2.5 (L)	**<0.001**	−5.7	−14.4; 3.0	0.194	0.389
Serum sodium_-30_ > 145 versus ≤145 (mmol/L)	**<0.001**	1.9	−6.9; 10.6	0.665	0.545
Serum potassium_-30_ > 5.0 versus ≤5.0 (mmol/L)	**<0.001**	2.4	−6.3; 11.2	0.576	0.558
Dialysis vintage	**<0.001**			0.781	0.317
0, 1 versus 2 (years)		−3.9	−16.2; 8.4		
≥3 versus 2 (years)		−3.4	−15.7; 8.9		

SBP_-30_, systolic blood pressure at the beginning of dialysis (baseline); HR_-30_, heart rate at the beginning of dialysis (baseline); Vo%, relative volume overload; *p*-values < 0.05 are marked in bold.

**Table 5:** Dynamics of heart rate during hemodialysis in relation to selected clinical and dialysis parameters.

	Time Effect *p*-Value	Between Effect			Interaction with Time *p*-Value
		Difference from the Reference Value		*p*-Value	
		Mean	95% CI		
Males versus females	**<0.001**	2.0	−5.6; 9.7	0.594	0.992
Age ≥ 65 versus <65 (years)	**<0.001**	−2.2	−9.5; 5.1	0.543	0.334
Body mass index	**<0.001**			**0.020**	0.891
25.0–29.9 versus ≤24.9 (kg/m^2^)		9.4	1.1; 17.6		
≥30.0 versus ≤24.9 (kg/m^2^)		−1.1	−9.3; 7.1		
Diabetes—presence versus absence	**<0.001**	−9.6	−16.3; 3.0	**0.006**	0.404
SBP_-30_ > 140 versus ≤140 (mm Hg)	**<0.001**	−1.7	−9.0; 5.6	0.641	0.848
HR_-30_ < 60 versus 60–100 (bpm)	**<0.001**	−18.1	−24.6; −11.6	**<0.001**	0.691
Vo% >15 versus ≤15 (%)	**0.019**	−2.3	−9.7; 5.1	0.531	0.210
Ultrafiltration volume ≥ 2.5 (L)	**<0.001**	2.9	−4.5; 10.2	0.436	0.649
Serum sodium_-30_ > 145 versus ≤145 (mmol/L)	**<0.001**	−2.8	−10.0; 4.5	0.440	0.465
Serum potassium_-30_ > 5.0 versus ≤5.0 (mmol/L)	**<0.001**	−1.2	−8.5; 6.1	0.748	0.493
Dialysis vintage	**<0.001**			0.456	0.385
0, 1 versus 2 (years)		−4.8	−13.4; 3.8		
≥3 versus 2 (years)		−4.4	−13.1; 4.2		

SBP_-30_, systolic blood pressure at the beginning of dialysis (baseline); HR_-30_, heart rate at the beginning of dialysis (baseline); Vo%, relative volume overload; *p*-values < 0.05 are marked in bold.

**Table 6:** Dynamics of pulse pressure during hemodialysis in relation to selected clinical and dialysis parameters.

	Time Effect *p*-Value	Between Effect			Interaction with Time *p*-Value
		Difference from the Reference Value		*p*-Value	
		Mean	95% CI		
Males versus females	**<0.001**	−7.4	−19.5; 4.7	0.222	0.275
Age ≥ 65 versus <65 (years)	**<0.001**	2.2	−9.6; 13.9	0.712	0.817
Body mass index	**<0.001**			0.620	0.631
25.0–29.9 versus ≤24.9 (kg/m^2^)		−0.5	−15.4; 14.4		
≥30.0 versus ≤24.9 (kg/m^2^)		5.6	−9.1; 20.2		
Diabetes—presence versus absence	**<0.001**	14.7	3.9; 25.5	**0.009**	**0.047**
SBP_-30_ > 140 versus ≤140 (mm Hg)	**<0.001**	20.1	10.6; 29.5	**<0.001**	0.693
HR_-30_ < 60 versus 60–100 (bpm)	**0.001**	13.3	−0.6; 27.3	0.060	0.127
Vo% > 15 versus ≤15 (%)	**<0.001**	13.5	2.2; 24.8	**0.021**	**0.005**
Ultrafiltration volume ≥ 2.5 (L)	**<0.001**	3.7	−8.3; 15.6	0.535	0.351
Serum sodium_-30_ > 145 versus ≤145 (mmol/L)	**<0.001**	0.5	−11.3; 12.2	0.938	0.108
Serum potassium_-30_ > 5.0 versus ≤5.0 (mmol/L)	**<0.001**	−0.4	−12.2; 11.4	0.948	0.565
Dialysis vintage	**<0.001**			0.673	0.490
0, 1 versus 2 (years)		−6.7	−22.4; 9.0		
≥3 versus 2 (years)		−2.3	−18.0; 13.4		

SBP_-30_, systolic blood pressure at the beginning of dialysis (baseline); HR_-30_, heart rate at the beginning of dialysis (baseline); Vo%, relative volume overload; *p*-values < 0.05 are marked in bold.

**Table 7:** Dynamics of mean arterial pressure during hemodialysis in relation to selected clinical and dialysis parameters.

	Time Effect *p*-Value	Between Effect			Interaction with Time *p*-Value
		Difference from the Reference Value		*p*-Value	
		Mean	95% CI		
Males versus females	**<0.001**	3.3	−5.8; 12.4	0.467	0.280
Age ≥ 65 versus <65 (years)	**0.001**	−6.1	−14.6; 2.4	0.152	0.954
Body mass index	**0.001**			0.454	0.485
25.0–29.9 versus ≤24.9 (kg/m^2^)		6.6	−4.3; 17.6		
≥30.0 versus ≤24.9 (kg/m^2^)		2.2	−8.6; 13.0		
Diabetes—presence versus absence	**<0.001**	−6.1	−14.8; 2.5	0.159	**0.020**
SBP_-30_ > 140 versus ≤140 (mm Hg)	**0.001**	7.3	−1.1; 15.6	0.086	0.959
HR_-30_ < 60 versus 60–100 (bpm)	**<0.001**	−2.2	−13.1; 8.7	0.681	**0.001**
Vo% >15 versus ≤15 (%)	**<0.001**	0.2	−9.2; 9.6	0.971	0.738
Ultrafiltration volume ≥ 2.5 (L)	**<0.001**	3.7	−8.3; 15.6	0.308	0.172
Serum sodium_-30_ > 145 versus ≤145 (mmol/L)	**0.001**	2.0	−6.7; 10.7	0.638	0.692
Serum potassium_-30_ > 5.0 versus ≤5.0 (mmol/L)	**0.001**	2.3	−6.4; 11.0	0.595	0.471
Dialysis vintage	**0.001**			0.590	0.520
0, 1 versus 2 (years)		−6.1	−18.4; 6.3		
≥3 versus 2 (years)		−4.2	−16.5; 8.2		

SBP_-30_, systolic blood pressure at the beginning of dialysis (baseline); HR_-30_, heart rate at the beginning of dialysis (baseline); Vo%, relative volume overload; *p*-values < 0.05 are marked in bold.

## Data Availability

The data presented in this study are available on request from the corresponding author. The data are not publicly available due to Polish General Data Protection Regulation.
